# Estimating Accessibility Problems in the Swedish Housing Stock Using Citizen Science: The Housing Experiment 2021

**DOI:** 10.1177/07334648241262646

**Published:** 2024-07-18

**Authors:** Björn Slaug, Marianne Granbom, Susanne Iwarsson

**Affiliations:** 1 Department of Health Sciences, Faculty of Medicine, 5193 Lund University, Sweden

**Keywords:** older adults, housing accessibility, environmental barriers, citizen science, aging-in-place

## Abstract

Accessible housing for the aging population is important, but large-scale reliable information on accessibility problems in ordinary housing is lacking. This study aimed to describe the prevalence of environmental barriers and analyze potential accessibility problems in the Swedish housing stock and to evaluate the validity and representativeness of housing data collected in a citizen science project. Data on environmental barriers in 1181 dwellings were collected by members of the public. Prevalence of barriers and potential accessibility problems were analyzed using descriptive statistics and ranking methodology. Validity and representativeness were addressed by comparisons with public statistics and research, and analysis of data properties. It was found there are substantial numbers of environmental barriers in dwellings across Sweden that generate accessibility problems for people with functional limitations. The results suggest that with user-friendly data collection tools and instructions, data validity and representativeness can be achieved in citizen science projects involving older adults.

**What this paper adds**

• Knowledge about involvement of older adults in a citizen science project targeting housing issues
• Knowledge about environmental barriers and accessibility problems in the ordinary Swedish housing stock
• Understanding of issues of validity and data quality in citizen science projects

**Applications of study findings**

• Citizen science projects should be considered as a viable way to involve older adults in data collection on issues of importance to them and society overall
• Citizen science projects need to include considerable methodological efforts to achieve data of high quality and validity
• Housing accessibility for the aging population should be further addressed both in research and by policymakers

## Introduction

Despite progressive population aging, housing provision meeting the needs of people as they age is lagging (see, e.g., Schekler et al., 2022), and there is an urgent need to better understand and provide for the diversity of housing requirements of older adults (Vespa et al., 2020). For example, even if accessible housing for people with functional limitations is a key issue for active and healthy aging (Jonsson et al., 2021; WHO, 2018), in most countries the ordinary housing stock does not meet even basic accessibility requirements. By accessible housing we mean housing designed or adapted to support activity and participation, despite reduced functional capacity. Earlier research indicates that inaccessible housing may affect autonomy (Haak et al., 2007), independence (Mahler et al., 2014), and participation (Thordardottir et al., 2018) in later life. Moreover, there is evidence that poor housing accessibility may increase the risk of fall accidents (Hu et al., 2015), mental health issues (Howden-Chapman et al., 2011), as well as mortality rates (Rantakokko et al., 2013).

For complex, societal challenges where any potential solution is only temporary and can always be contested, the term wicked problems has been suggested (Rittel & Webber, 1973). Considering that decades of public debate and policy ambitions to deal with providing accessible housing for the aging population have not resulted in any decisive solutions, this issue bears the characteristics of a wicked problem (Jonsson et al., 2021). Wicked problems are characterized by involving different dimensions and multiple critical variables in an intertwined manner, that have to be analyzed and considered from sometimes conflicting perspectives and interests of several actors. A basic dimension in addressing a wicked problem concerns how the issue is measured and quantified. Reflecting on this dimension, there is a lack of systematic information about the state of accessibility in the housing stock, with scarce knowledge on how person-environment interactions in later life affect housing accessibility. In accordance with the Ecological Theory of Aging and the concept of person-environment fit (Lawton & Nahemow, 1973), individuals with reduced functional capacity are more sensitive to the demands of the physical environment. However, a good person-environment fit can still be achieved by lowering the environmental demands. Better knowledge of the impact of specific housing design features on person-environment fit in later life is therefore critical in improving housing accessibility. Dealing with housing provision for the aging populations, there is also an ethical dimension that relates to equality, personal responsibility and freedom of choice that is necessary to consider. Another important dimension is the empathic dimension, which is critical for communication striving to understand and influence attitudes and feelings about housing accessibility. Jonsson et al.’s (2021) findings indicate that empathetic and creative ways to influence attitudes are vital to changing people’s positions and bridging boundaries.

For more than 20 years we have collected reliable and detailed data on environmental barriers and housing accessibility in several countries (see, e.g., Iwarsson et al., 2006). The largest existing dataset contains data collected by researchers at home visits with different samples of older adults in southern Sweden, which has been used to extrapolate the findings to the national level (Granbom et al., 2016). The findings showed that high proportions of dwellings had substantial numbers of environmental barriers, albeit with significantly lower numbers in later building periods. However, as 75% of the current Swedish housing stock was built before the 1980s when guidelines and building regulations concerning accessibility were introduced, most of the older segment of the population lives in dwellings with substantial accessibility problems (Table 1). Noteworthy, the study by Granbom et al. (2016) showed there were considerable accessibility problems already for older adults with few functional limitations, and markedly more prominent especially for people using mobility devices. In single-family dwellings the entrance was the most problematic housing section, while the kitchen was the most problematic in multi-family dwellings.

**Table 1.** Distribution of the Swedish Ordinary Housing Stock 2021.^a^

Building period	Single-family house % (*n*)	Multi-dwelling block % (*n*)	Total % (*N*)
−1960	41 (861 967)	39 (1 014 992)	40 (1 876 959)
1961–1980	34 (719 121)	34 (882 813)	34 (1 601 934)
1981–2010	20 (430 754)	17 (436 736)	18 (876 490)
2011-w	5 (94 237)	11 (287 190)	8 (381 427)
Total	100 (2 106 079)	100 (2 621 731)	100 (4 727 810)

*Note.* Building year was unknown for 14,356 dwellings in the Swedish ordinary housing stock 2021.

^a^Statistics Sweden (2023).

For a long time in Sweden, housing accessibility has been promoted for by senior citizen organizations and advocates for the disability rights movement, though hitherto mainly on the national policy level. Tapping into these efforts to promote housing accessibility, a citizen science project was initiated together with the non-governmental organization Science & Public, with older adults and people with functional limitations as the main target groups—The Housing Experiment 2021. Citizen science means scientific research conducted in whole or in part by members of the public (Heiss & Matthes, 2017; Kasperowski & Brounéus, 2016). It serves the purposes both of gaining scientific insights and knowledge that would be difficult or impossible for professional researchers to gain alone, and to engage the public in scientific activities. With the empathic dimension of the wicked problem of providing accessible housing for the aging population in mind, the ambition of this project was to incentivize members of the public to collect detailed data of environmental barriers in the ordinary housing stock on a large scale, by means of a user-friendly app using research-based questions (Granbom et al., 2023). Underlying ideas of the project were also to engage public citizens in research activities (Wood et al., 2022), and to evaluate their capacity as co-researchers and explore the potential effects on attitudes to housing accessibility (Frögren et al., 2023) as an avenue towards local actioning.

In any research project it is critical to assure the highest possible quality of data, and in academic research there are usually extensive resources invested for that purpose. However, this is not often the case in citizen science, and in a recent study data quality was identified as “the Achilles heel of citizen science projects” (Balazs et al., 2021). This is manifested, for instance, in ambiguous definitions of data quality and unclear procedures of how to achieve it. Data quality issues are also a reason why citizen science projects now and then have been viewed with skepticism and distrust from both researchers and policymakers (Bonney et al., 2014; Kosmala et al., 2016; Nascimento et al., 2018). To gain credibility for citizen science projects it is therefore important to scrutinize data collected by members of the public and to evaluate quality in a transparent manner.

Based on the empirical data collected during the Housing Experiment 2021, the aim of the current study was twofold. First, we aimed to describe the prevalence of environmental barriers in the housing stock in Sweden, and to analyze accessibility problems by relating the data to combinations of functional limitations that are common in the aging population. Second, we aimed to evaluate the validity and representativeness of housing data collected by members of the public.

## Material and Methods

### The Housing Experiment App

The Housing Enabler (HE), which is a research-based instrument for assessing and analyzing housing accessibility problems in terms of person-environment fit (Carlsson et al., 2009; Iwarsson et al., 2012) constituted the scientific base for the data collection in the Housing Experiment 2021. The HE comprises an environmental (physical barriers) and a personal (functional limitations) component that are combined to quantify potential accessibility problems. The environmental component of the HE consists of 161 potential environmental barriers in and around the home, based on standards and guidelines for housing design as expressed in national legislation and guidelines. For screening purposes, a shortened list of 60 environmental barrier items that are most important to detect housing accessibility problems on population or group level was launched (Carlsson et al., 2009). Using this shortened environmental checklist of the HE as the starting point, 33 items were selected and adapted into a citizen science version of the HE, in a rigorous process (described in detail elsewhere, see Granbom et al., 2023). Focusing on the entrance and indoor environment, items concerning the exterior surroundings (20 items) were not included in the citizen science version, and other items were excluded mainly due to very high prevalence (>95%) confirmed in previous research. Moreover, during this process, some items were split into more than one question, and some were repeated for different locations in the dwelling, resulting in a total of 40 questions in the citizen science version of the HE. However, to make the current study comparable to previous research using the original instrument, the split questions were recombined in the analysis and presentation of results.

**Figure 1.**
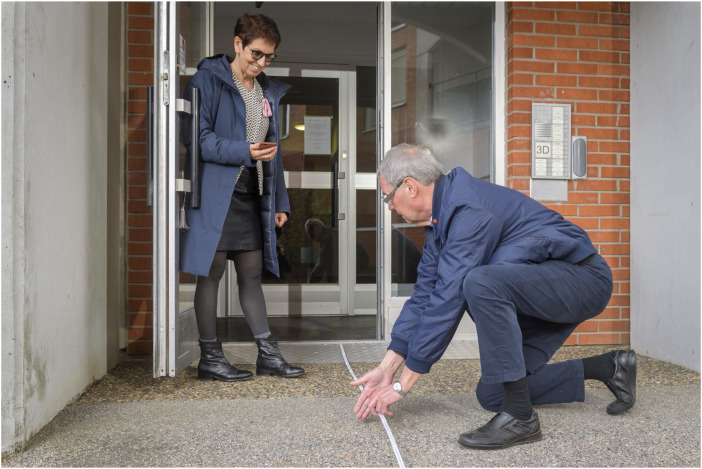
Conducting measurements to answer questions in the Housing Experiment app. Photo by Kennet Ruona.

For members of the public to collect data, together with the non-profit organization Science & Public we developed a web application (app) for IOS and Android for use on smartphones and tablets. The app was developed in an iterative development process in six phases including participatory activities with researchers, citizen science specialists, tech developers, older adults, and other members of the public. Substantial efforts were made to ensure that the app was easy to use regardless of digital literacy. After usability testing involving older adults that lead to some adjustments and fine-tuning of the interface, the app was evaluated as usable and reliable (Granbom et al., 2023). The app was open to download for free on Google Play and App Store. All instructions needed were embedded in the app. Supporting information was published online in text and video. Although the main efforts were directed at older adults as the target group, the intention was to make it available for all in the public with an interest in participating as data collectors (Figure 1).

### Data and Data Collection

Descriptive questions in the app were used to collect address zip code and information about type of dwelling, building year, and major renovations. Questions about the residents in the dwelling relevant for accessibility issues, that is, if any of them were 65–79 years old; 80 years or older; used a wheeled walker or wheelchair in the dwelling followed. The 40 Yes/No questions derived from the HE addressed the presence of specific design features at the entrance, in the kitchen, bathroom, and in other parts of the dwelling. For example, the user had to respond Yes/No to questions such as “Do all flights of stairs have bannisters on both sides?”, and measure with a ruler to respond Yes/No to questions such as “Are any of the thresholds one needs to pass at the entrance higher than 15 millimeters?” It was possible for the user to skip questions and still register their responses. Upon completion the responses registered in the app were automatically uploaded to a database, which was updated in real-time on a public project website (The Housing Experiment 2021). Data was collected during 2.5 months in fall 2021.

### Recruitment of Participants for Data Collection

Information about the citizen science project and recruitment efforts were made by all partners of the Housing Experiment 2021. Various local and national news outlets were contacted, which resulted in about 20 press clippings, two broadcasts on national radio and one on a national TV channel, with a total reach of 8,637,496 persons. Science & Public informed their members by internal advertising, media coverage in member magazines or directly via email to members and noted more than 80 posts on social media with more than 34,000 impressions. Interest associations focusing on issues relating to either older adults, people with disabilities or housing (such as tenants' and housing company associations) were approached and offered written and video material to use for information about the project. In addition, partnering associations used social media to inform about the Housing Experiment 2021 and attract participants. In line with previous citizen science projects involving the Science & Public organization, there was also an effort to reach teachers at public schools and offer their pupils the possibility to engage in data collection. Although special curriculum-adapted material was also provided, there was however little interest in the public schools to engage in this project. In all, 1181 members of the public participated in the data collection (70% aged 65 or older).

### Data Analysis

Descriptive statistics (percentages or median and quartiles) were used to present basic characteristics of the dwellings, and to present number of environmental barriers (min–max = 0–33) by dwelling type and building period. For comparisons of prevalence of specific barriers between dwelling type and building periods, chi-square test was used. All calculations were based on valid answers.

To analyze the barriers generating most accessibility problems at group level we used functional profiles defined through previously reported simulations and statistical analyses of large data sets on functional limitations of older adults (Granbom et al., 2016; Slaug et al., 2011). Four functional profiles with increasing complexity were used: Profile I: limitations in movement only; Profile II: limitations in movement and upper extremity; Profile III: limitations in movement, upper extremity and dependence on mobility devices; and Profile IV: limitations in movement, upper extremity, dependence on mobility devices and loss of sight. For each environmental barrier an accessibility problem score based on the HE methodology was calculated (Slaug et al., 2011). The accessibility problem score is calculated by combining the environmental items with the functional profiles, using a scoring matrix with pre-defined severity ratings (0–4) that are summed up to a total score, which represents a quantification of predicted accessibility problems. Next, ranking lists of environmental barriers in descending order according to the magnitude of accessibility problems generated were produced.

To evaluate validity and representativeness of the data the distributions of type of dwelling and building period were compared to publicly available national statistics (Statistics Sweden, 2023). The ranking order of barriers generating the most accessibility problems was compared to previous research results based on existing data collected by trained data collectors at home visits, extrapolated to the national level (Granbom et al., 2016). To further evaluate validity, we calculated the missing ratio for each of the 40 questions concerning the presence of environmental barriers, considering a missing ratio of at most 5% as acceptable We tested internal data consistency based on the assumption that there is pattern in the distribution of barriers and accessibility problems at national level that should be consistent throughout the Housing Experiment dataset. To test this, we applied a type of split-half procedure (Carmines & Zeller, 1979). That is, we first randomly split the data in two equal subsets, and then tested for differences in the accessibility problem scores for the four functional profiles between the two subsets using Kruskal–Wallis test.

The SAS Software, SAS Institute Inc., Cary, NC, Version 9.4 was used for all analyses.

*p*-values <0.05 were considered statistically significant.

## Results

The total number of dwellings assessed was *N* = 1181. Type of dwelling was evenly divided between single-family houses (51%) and apartments in multi-dwelling blocks (49%). Regarding building year, the period of the million homes program in Sweden (i.e., 1960–1979) dominated (36%). Ten percent of the dwellings were built after 2010. In 15% of the dwellings there were residents aged 80+ and in 10% there were residents using mobility devices. For details, see Table 2.

**Table 2.** Basic Characteristics of Dwellings Assessed by Members of the Public, *N* = 1181.

Variable	% (*n*)	Missing *n*
Type of dwelling		10
Single-family house	51 (592)	
Multi-dwelling block	49 (579)	
Building period		39
<1960	28 (317)	
1960–1979	36 (415)	
1980–2010	26 (296)	
2011-w	10 (114)	
Renovated	28 (304)	90
Individual using mobility devices living in dwelling	10 (119)	22
Individual aged 80+ living in dwelling	15 (170)	35

In total, there were more environmental barriers in single-family houses (median = 16, q1-q3 = 13–19) compared to in apartments in multi-dwelling blocks (median = 13, q1-q3 = 9–16). The highest number of barriers was identified at entrances (median = 5, q1-q3 = 3–6). As to building period, there were fewer barriers in newer dwellings, particularly notable for barriers at entrances (median = 2, q1-q3 = 1–4). See Table 3 for details.

**Table 3.** Number of Environmental barriers by Type of Dwelling and by building Period, *N* = 1181.

	Type of dwelling^a^		Building period^b^				Total *N* = 1181
	Single-family house, *n* = 592	Multi-dwelling, *n* = 579	<1960 *n* = 317	1960–1979 *n* = 415	1980–2010 *n* = 296	2011-*n* = 114	
	Median (quartile 1–quartile 3)						
Total number of barriers	16 (13–19)	13 (9–16)	16 (14–19)	15 (12–19)	12 (9–15)	9 (6–12)	14 (11–18)
Entrance	5 (4–6)	4 (2–6)	6 (4–7)	5 (3–6)	4 (2–5)	2 (1–4)	5 (3–6)
Kitchen	2 (2–3)	2 (2–3)	2 (2–3)	2 (2–3)	2 (2–3)	2 (1–3)	2 (2–3)
Bathroom	4 (3–4)	4 (3–4)	4 (3–5)	4 (3–4)	3 (3–4)	3 (3–4)	4 (3–4)
Other location	4 (2–7)	3 (1–4)	4 (2–6)	4 (3–6)	2 (1–3)	2 (0–2)	3 (2–5)

^a^Information about type of housing missing for 10 cases.

^b^Information about building period missing for 39 cases. Total number of barriers = 0 to 33.

No grab bar at shower and/or toilet was the most frequent barrier overall (92%), consistently with a prevalence >90% across building periods. However, in single-family houses stairs the only route at entrance was even more frequent (98%). Significant differences between single-family houses and apartments in multi-dwellings blocks concerned a higher number of thresholds, both at entrances and indoor locations in single-family houses (*p* < .001). Barriers at entrances, such as stairs the only route, thresholds, steps and short handrails were significantly less prevalent in later building periods (*p* < .001). See Tables 4 and 5 for details.

**Table 4.** Most Prevalent Environmental barriers by Type of Dwelling, *N* = 1181.

Location	Environmental barrier	Total % (*n*)	Single-family house % (*n*)	Multi-dwelling block % (*n*)	*p*-value^a^
Bathroom	No grab bar at shower and/or toilet	92 (1086)	95 (560)	89 (518)	**.001**
Kitchen	No surface suitable for sitting while working	87 (1029)	89 (525)	86 (498)	.169
Entrance	Stairs the only route	83 (982)	98 (578)	68 (396)	**<.001**
Bathroom	Wash basin at a height only for standing	83 (981)	82 (487)	84 (487)	.398
Other location	High threshold/level difference/step	76 (899)	83 (489)	70 (406)	**<.001**
Bathroom	Toilet 47 cm or lower	75 (880)	75 (443)	74 (431)	.877
Other location	Thresholds/level differences/steps between rooms	67 (796)	73 (434)	61 (354)	**<.001**
Entrance	Handrails too short	63 (736)	79 (466)	46 (264)	**<.001**
Entrance	High threshold and/or steps at entrance	55 (646)	71 (422)	38 (218)	**<.001**
Kitchen	Shelves too deep	54 (636)	52 (306)	56 (322)	<.178

*Note.* Information about type of housing missing for 10 cases.

^a^Chi-square test. Significant p-values are bolded.

**Table 5.** Most Prevalent Environmental barriers by building Period, *N* = 1181.

Location	Environmental barrier	Total % (*n*)	<1960	1960–1970	1980–2010	2011-Present	*p*-value^a^
Bathroom	No grab bar at shower and/or toilet	92 (1086)	93 (296)	91 (379)	92 (272)	91 (104)	.760
Kitchen	No surface suitable for sitting while working	87 (1029)	85 (270)	88 (365)	91 (269)	88 (100)	.196
Entrance	Stairs the only route	83 (982)	93 (294)	86 (357)	75 (222)	58 (66)	**<.001**
Bathroom	Wash basin at a height only for standing	83 (981)	82 (261)	84 (347)	82 (244)	86 (99)	.699
Other location	High threshold/level difference/step	76 (899)	72 (229)	78 (323)	83 (246)	52 (59)	**<.001**
Bathroom	Toilet 47 cm or lower	75 (880)	74 (234)	76 (315)	72 (212)	76 (87)	.583
Other location	Thresholds/level differences/steps between rooms	67 (796)	78 (246)	75 (310)	57 (169)	32 (37)	**<.001**
Entrance	Handrails too short	63 (736)	68 (257)	68 (284)	48 (142)	25 (29)	**<.001**
Entrance	High threshold and/or steps at entrance	55 (646)	63 (200)	58 (242)	49 (144)	36 (41)	**<.001**
Kitchen	Shelves too deep	54 (636)	59 (188)	53 (221)	57 (170)	35 (40)	**<.001**

*Note.* Information about building period missing for 39 cases.

^a^Chi-square test. Significant p-values are bolded.

The barriers generating most accessibility problems across the four profiles of functional limitations were lack of grab bars in bathrooms, shelves placed too high in kitchens, and stairs the only route at entrances. The ranking patterns were fairly consistent across the functional profiles. In multi-dwelling blocks accessibility problems generated by barriers related to entrance doors (e.g., heavy door without automatic opener; door that does not stay in open position/close quickly) were prominent in terms of ranking, while stairs to upper story or basement with necessary functions were more prominent as a generator of accessibility problems in single-family houses (Table 6).

**Table 6.** Rank Order of the Environmental barriers Generating Most Accessibility Problems, by Functional Profile (I–IV) and Housing Type.

Environmental barrier (*n* = 33)	Multi-dwelling block (*n* = 579)				Single-family house (*n* = 592)			
	Profile I^a^	Profile II^b^	Profile III^c^	Profile IV^d^	Profile I^a^	Profile II^b^	Profile III^c^	Profile IV^d^
Entrances								
Narrow door openings	e	e	24	27	e	e	29	30
High thresholds and/or steps at the entrance	**8**	**10**	12	**9**	**7**	11	**6**	**6**
Insufficient maneuvering space at doors	e	e	16	16	e	e	12	13
No resting area in front of entrance door	e	e	23	26	e	e	20	24
Heavy doors without automatic door opener	**6**	**3**	**4**	**7**	14	**7**	15	17
Doors do not stay in open position/close quickly	**5**	**7**	**9**	**6**	22	22	28	27
Complicated/illogical opening procedure	19	12	21	18	25	23	31	29
Stairs the only route (no lift/ramp)	**3**	**5**	**3**	**3**	**2**	**3**	**2**	**1**
High, low or irregular heights of risers	17	18	29	23	12	15	26	18
No handrails or only on one side	12	14	20	19	**4**	**5**	11	9
Handrails too short	15	16	22	21	15	17	22	14
Kitchen								
Insufficient maneuvering space around white goods	11	**9**	14	11	17	13	18	12
Shelves placed too high	**2**	**1**	**2**	**1**	**3**	**1**	**3**	**3**
No surface at height suitable for sitting while working	13	15	**8**	**10**	18	19	**8**	11
Shelves too deep	14	4	**5**	**8**	19	**4**	**7**	**10**
Complex maneuvers and good precision required	26	24	32	32	26	26	32	32
Bathroom								
Insufficient maneuvering space where turning is required	18	19	**10**	12	21	21	14	16
Insufficient space for stool/bath board or equivalent	23	23	28	29	24	25	30	31
No grab bar at shower/bath or toilet	**1**	**2**	**1**	**2**	**1**	**2**	**1**	**2**
Complex maneuvers and good precision required	27	25	33	33	27	27	33	33
Wash basin at height only for standing	**9**	11	13	14	13	16	17	21
Toilet 47 cm or lower	**10**	13	11	13	16	18	13	15
Shower stall with kerb/level difference	22	22	25	28	20	20	25	26
Bathtub instead of shower space	16	17	19	22	23	24	27	28
Other areas								
Narrow doors to sitting-out place/balcony	e	e	18	20	e	e	19	25
High threshold/level difference/step to sitting-out place balcony	**4**	**6**	**6**	**4**	**5**	**6**	**4**	**4**
Steps/thresholds/difference in level between rooms	**7**	**8**	**7**	**5**	**6**	**10**	**5**	**5**
Narrow passages/corridors in relation to fixtures	e	e	15	15	e	e	16	19
Narrow doors	e	e	17	17	e	e	24	23
Stairs to upper story with necessary functions	20	20	26	24	**8**	**8**	**9**	**7**
Stairs to basement with necessary functions	20	20	26	24	**8**	**8**	**9**	**7**
Handrails or only on one side	24	26	31	31	**10**	12	21	20
Handrails too short	25	27	30	30	11	14	23	22

*Note.* Top-10 barriers of each functional profile and housing type are bolded (10 cases had missing information of housing type).

^a^Profile I: Functional profile with limited mobility.

^b^Profile II: Functional profile with limited mobility and limited upper extremity function.

^c^Profile III: Functional profile with limited mobility, limited upper extremity function, and dependence on mobility devices.

^d^Profile IV: Functional profile with limited mobility, limited upper extremity function, dependence on mobility devices, and visual impairment.

^e^Did not generate any accessibility problem score.

Comparing the results with available national statistics, single-family houses were slightly overrepresented in our sample (51% vs. 45%). There were fewer dwellings built before 1960 in our sample (28% vs. 40%), while the proportion of dwellings from the period of the million homes program was similar to the national housing stock (36% vs. 34%). The barriers generating most accessibility problems were very similar to our previous research. That is, lack of grab bars in bathrooms, shelves placed too high in kitchens and stairs the only route at entrances generated most accessibility problems. In apartments in multi-dwelling blocks, heavy doors without automatic opener and door that does not stay in open position/close quickly were equally prominent in our sample as in our previous research, as were stairs to upper story or basement with necessary functions in single-family houses. The ratio of missing values for the 40 questions concerning the presence of environmental barriers ranged from 0.8% to 1.2%. Test of internal data consistency showed no significant difference between the two randomly split subsets (*p*-value range = 0.228–0.384).

## Discussion

The results of the Housing Experiment 2021 largely confirmed what previous research has found, that is, there are substantial numbers of environmental barriers in the ordinary housing stock in Sweden (Granbom et al., 2016; Pettersson et al., 2018), both in single-family houses and apartments in multi-dwelling blocks. These environmental barriers generate accessibility problems for many older adults. Evaluating the data collected by members of the public, the results suggest that high data quality, validity and representativeness can be achieved in a citizen science project with older adults as the main target group.

The high prevalences of environmental barriers (70–90%) such as no grab bar in bathroom, stairs the only route to enter the dwelling, thresholds, steps or level differences between rooms, kitchen shelves too deep and lack of surfaces suitable for sitting while working in the kitchen are serious because such barriers generate accessibility problem for people with functional limitations (see Table 6). For thresholds, steps or level differences between rooms there was a notable decrease in prevalence over time (32% in the newest dwellings), but for lack of surfaces suitable for sitting while working in the kitchen (88% in the newest dwellings) and lack of grab bars in bathroom (91% in the newest dwellings) there was no change over time at all. To take the consequences of the aging population for accessible housing better into account, the functional profiles based on person-environment fit calculations used in this study are important. That is, with the aging population, there is an ongoing increase of older adults with more complex combinations of functional limitations, such as both mobility and sensory limitations. For instance, the results of the present study suggest that for multi-dwelling blocks, problems related to the entrance, such as heavy doors without automatic opener and doors that do not stay open or close quickly, generate serious accessibility problems and should have high priority even though they are not the most prevalent. Concerning single-family houses, accessibility problems related to necessary functions located at upper stories or in the basement stand out in a similar manner. On a note of caution, the comparison with previous research concerning accessibility problems was based on ranking order in terms of severity of problems for different functional profiles and not the accessibility problem scores as such. Still, the ranking order identified in the present study was found to be consistent with previous research (Granbom et al., 2016).

While many accessibility problems could be avoided by more foresighted design when building new housing and more efficient home modification strategies (Kim et al., 2024; Sheth & Cogle, 2023), these results indicate an alarming lack of action in the housing provision sector. Already 30 years ago, considerable accessibility issues for older people in the Swedish ordinary housing stock were highlighted in public health research (Iwarsson & Isacsson, 1993). Moreover, in 2015 there was an investigation from the Swedish government (SOU, 2015) that suggested several measures to upgrade the ordinary housing stock in terms of physical accessibility, against the background of the aging population. Considering the results of the current study there are no indications that those measures have come into effect, which further underlines the urgency for all actors in the housing provision sector to take joint action to address the issue of accessible housing in a constructive manner.

To understand the longstanding lack of action in a matter of such social urgency that has from time to time even been high on the political agenda, the perspective of wicked problems (Rittel & Webber, 1973) is useful. Although many actors in the housing provision sector seemingly agree that housing accessibility for the aging population is important to address (Jonsson et al., 2021), typical for wicked problems there is not a common understanding of how to define the issue, how best to deal with it and how to determine when it has been sufficiently addressed (Rittel & Webber, 1973). This suggests that for joint actions to materialize in concrete efforts, economic incentives for all actors need to be strengthened and building legislation has to be both sharper and more specific in the requirements. Recent studies and reports in the US also highlight that current programs such Medicaid home and community-based care waivers are narrow and entirely insufficient to deal with the rapidly growing issue of providing accessible housing for the aging population (Schekler et al., 2022; Vespa et al., 2020).

The similarities of the results of this study and publicly available statistics and research as well as the low missing ratios and the internal consistency of data suggest that the Housing Experiment 2021 produced results that are valid and fairly representative for the Swedish ordinary housing stock. A key factor behind this achievement is the participatory methods applied during the development process (Granbom et al., 2023). Representatives of the targeted users had a direct impact when developing the appearance and functions of the user interface. In addition, older adults were engaged in usability tests of the app prototype, and their feedback helped to fine-tune the final version. Due to a presumed lower level of digital literacy among older adults (Frögren et al., 2023), we took extra care to ensure acceptance and user-friendliness of the digital data collection tool, adding helpful instructions and guidance how to assess and enter data. Thus, the efforts made to make the app easy to use, and the outreach to a broad audience using a variety of communication channels appear to have paid off. The low missing ratios further indicate that the app was successful in this aspect.

Scrutinizing the underlying prevalence figures for specific barriers (Tables 4 and 5), they appear to be at the higher end of the spectrum compared to other previous studies (see, e.g., Pettersson et al., 2018). This might indicate that the Housing Experiment participants tended to be those who already had some awareness of the presence of environmental barriers in their homes and therefore were more interested in these issues, and thus, to engage in the citizen science project. The results of a recent study support this reasoning (Frögren et al., 2023). It should be kept in mind though, that this does not diminish the validity of the results; the ranking order of environmental barriers generating accessibility problems is valid and highlights the most significant issues. However, some statistical adjustment for overrepresentation of dwellings with common barriers might be necessary to estimate the true prevalences. Building on methodological knowledge from the current project, additional initiatives attracting larger groups of citizens to be engaged in data collection would allow the use of “Big Data” methods from zoological and ecological projects to further account for variability, representativity and data quality (Brown & Williams, 2019; Kelling et al., 2015).

With the citizen science version of the HE, we have developed and used a lay person’s version of an instrument originally developed for research and professional practice purposes. To the best of our knowledge, there are few such examples. Though the HE has been used in several large research projects over the years (Iwarsson et al., 2007; Kylén et al., 2014; Nilsson & Iwarsson, 2013), the level of detail and the complexity of the instrument makes it time-consuming and resource demanding, which has hampered a wider use of the instrument. With a citizen science version that captures the most important aspects of housing accessibility, the possibilities for use are significantly extended. For instance, municipalities can use the app to involve citizens to inventory dwellings to detect areas where accessibility problems require special attention. Citizens can also use it themselves to be better informed of potential future accessibility problems in their own dwellings, or to put pressure on housing companies, and political decision-makers to address these issues on the societal level (Wood et al., 2023). That is, the citizen science version of the HE has the potential to become a tool for empowerment of citizens in housing provision issues.

In conclusion, targeting all members of the public but with older adults as the main target group, the Housing Experiment 2021 produced valid results on environmental barriers and accessibility problems in the ordinary housing stock, contributing to accumulated knowledge in this research field. Further, the results suggest that with user-friendly data collection tools and instructions, data validity and representativeness can be achieved in citizen science projects involving older adults. A lesson learned is that considerable methodological efforts that must be carefully planned and implemented are necessary to achieve high quality data and valid results. Citizen science is an underutilized approach to involve older adults in data collection on issues of importance to them and society overall, and the Housing Experiment 2021 has potential to serve as inspiration for future citizen science projects.
